# Coronary intervention for acute coronary syndrome in a 51-year-old man with immune thrombocytopenic purpura: a case report

**DOI:** 10.1186/1752-1947-8-214

**Published:** 2014-06-20

**Authors:** Bora Demircelik, Meltem Altinsoy, Fadime Bozduman, Mahmut Gunes, Muzaffer Cakmak, Beyhan Eryonucu

**Affiliations:** 1 Department of Cardiology, Faculty of Medicine, Turgut Ozal University, Ankara, Turkey; 2 Department of Internal Medicine, Faculty of Medicine, Turgut Ozal University, Ankara, Turkey

**Keywords:** Acute coronary syndrome, Coronary intervention, Immune thrombocytopenic purpura

## Abstract

**Introduction:**

Treatment of the rare cases of patients with chronic idiopathic thrombocytopenic purpura with acute coronary syndrome can be a significant problem. The patient in our case report was treated successfully with percutaneous coronary intervention.

**Case presentation:**

A 51-year-old man of Turkish origin who had idiopathic thrombocytopenic purpura was admitted to our hospital with severe chest pain. His electrocardiography was normal on admission but dynamic ischemic changes were observed during follow-up. He underwent immediate coronary angiography. In his angiography, left anterior descending artery stenosis was 90% together with the diagonal ostium. Percutaneous coronary intervention was performed successfully. Bleeding complications were not observed after the procedure.

**Conclusions:**

We report the presence of a rare case of chronic idiopathic thrombocytopenic purpura in a patient with acute coronary syndrome. In this situation a serious multidisciplinary approach is required before coronary intervention.

## Introduction

Immune thrombocytopenic purpura (ITP) is the condition caused by increased platelet destruction and/or decreased platelet production following a viral infection. Clinical features associated with ITP are related to thrombocytopenia: petechiae (pinpoint microvascular hemorrhages that do not blanch with pressure), purpura (appearing like large bruises), epistaxis (nosebleeds), menorrhagia, gum bleeding, and other types of mucocutaneous bleeding. Other common clinical features include fatigue, impaired quality of life, and treatment-related side effects (for example, infection) [1]. Atherosclerosis and acute myocardial infarction (AMI) development are very infrequent in patients with chronic thrombocytopenia and congenital coagulopathies. Even in patients with AMI, thrombolytic therapy is contraindicated in those with immune thrombocytopenia [2]. Furthermore, the implementation of primary percutaneous coronary intervention (PCI) in these patients is rare [3,4]. In this case report we describe a patient who underwent primary PCI with chronic ITP and AMI without bleeding complications.

## Case presentation

**Figure 1:**
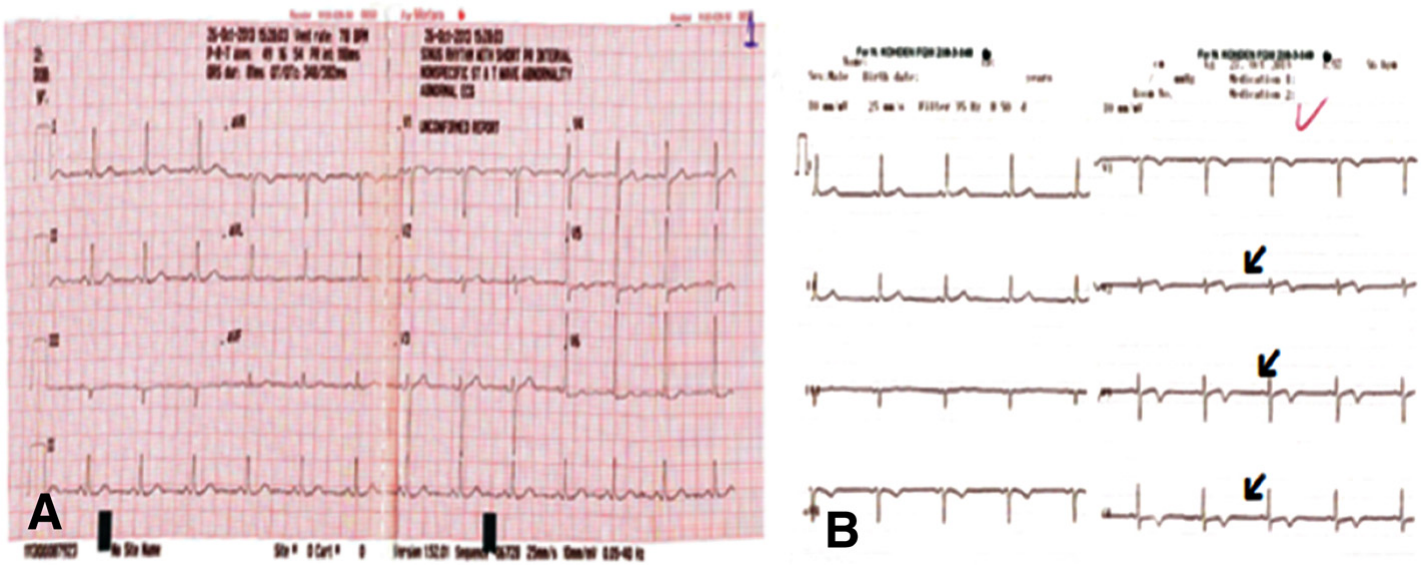
**Images of the patient’s electrocardiogram. A)** Electrocardiography on admission. **B)** Follow-up electrocardiographs (arrows indicate dynamic changes).

**Figure 2:**
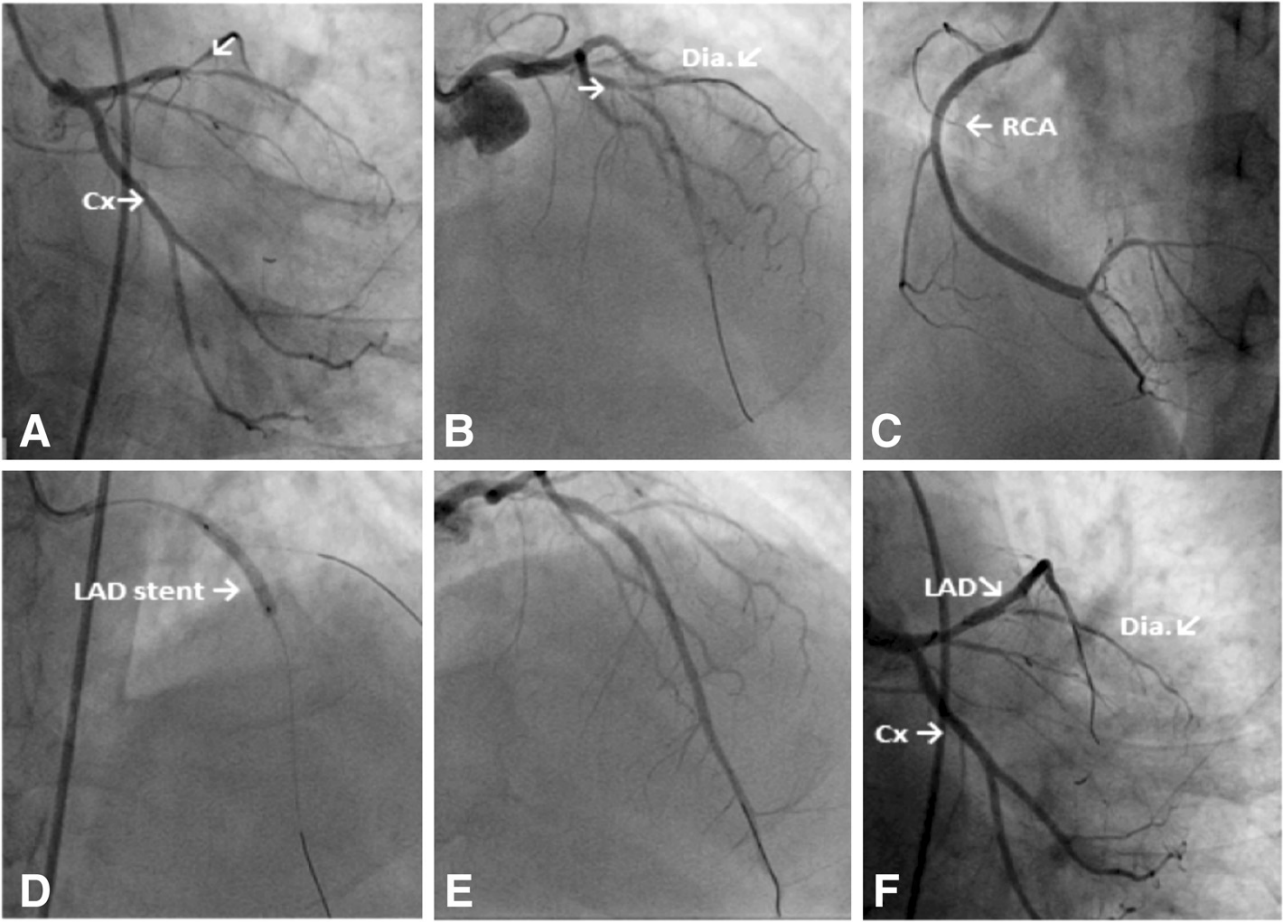
**Coronary angiography images. A)** Left anterior descending artery and circumflex images (right caudal). **B)** Left anterior descending artery and circumflex images (anterior cranial); arrow shows stenosis in left anterior descending artery. **C)** Right coronary artery image. **D)** Percutaneous coronary intervention of left anterior descending artery image. **E)** and **F)** The final version of left anterior descending artery after the procedure (right cranial –anterior caudal). Abbreviations: **Cx**, Circumflex; **Dia**, Diagonal artery; **LAD**, Left anterior descending artery; **RCA**, Right coronary artery.

A 51-year-old man of Turkish origin, who is a smoker, was admitted to our emergency department with retrosternal localized typical acute chest pain that started 1 day prior to presentation but was continuous during the last 20 minutes. A past diabetes mellitus, hypertension, dyslipidemia and family history were not found. His past medical history was significant for chronic ITP, for which he had not received any treatment, for 15 years. His physical examination was unremarkable, and neither cutaneous nor mucosal petechia nor purpura was noted. According to his electrocardiography (ECG) report there were biphasic T waves in anterior derivation (Figure 1A). In his transthoracic echocardiograph it was observed that his left ventricular apex septum and the middle of the anterior wall were hypokinetic. Laboratory blood tests revealed hemoglobin of 14mg/dL, platelet count of 14,000/μL, normal prothrombin time and partial thromboplastin time, troponin I of 1.89ng/mL, and creatine kinase MB 69U/L. Early coronary angiography and PCI were planned because of his continuous angina pectoris and dynamic ECG changing (Figure 1A, 1B). He received in the emergency room nitroglycerin and morphine as well as 300mg of clopidogrel and 325mg of aspirin along with 5000 units of heparin bolus. Prior to coronary angiography, one dose of intravenous immunoglobulin (IVIG) and six units of platelet transfusion were performed on the advice of the hematology department. Primary PCI was performed. A femoral approach was preferred; Allen test was positive in both his upper extremities. His right common femoral artery was accessed with a 6-French sheath. Diagnostic angiography revealed acute 90% thrombotic occlusion of the midsegment of his left anterior descending (LAD) artery and diagonal ostium. His left circumflex and right coronary arteries were patent with right system dominance (Figure 2A, 2B, 2C). A 6-French Judkins LAD 3.5 guiding catheter was used to intubate his left coronary system. An activated clotting time of 270 seconds was achieved. A 3.0×18mm bare metal stent (Gazelle, Medtronic, United States of America) was deployed in the midsegment of his LAD with optimal angiographic result and Thrombolysis In Myocardial Infarction grade flow 3 in his LAD artery (Figure 2D, 2E, 2F). During the operation his diagonal artery was protected with another wire. He was not given glycoprotein (Gp) IIb/IIIa inhibitors because of his low platelet number. The sheath introducer was removed after 6 hours with manual compression of the puncture site. His platelet number was between 57,000 and 63,000/μL in hospital. No bleeding or ischemic condition was observed. During his stay in hospital, he was given another dose of IVIG on the advice of the hematology clinic. He was discharged on aspirin 325mg and clopidogrel 75mg. His platelet level upon discharge was 159,000/μL. After 2 weeks, he has no symptoms and the movement of his left ventricular walls was found to be normal in his echocardiography.

## Discussion

ITP is a disease characterized by immunoglobulin G autoantibodies that bind to the surface of platelets leading to their phagocytosis in the reticuloendothelial system; this translates into thrombocytopenia and mucocutaneous bleeding [5]. Myocardial infarction may occur when platelets are low, and when it occurs a careful balance between usual anticoagulation and antiplatelet therapy on the one hand and efforts to raise platelet count on the other hand is needed. Acute coronary events have occurred in patients with ITP regardless of their platelet count which could range from normal to as low as 2×10^9^/L. Several incidences of myocardial infarction coincided with the administration of IVIG or rituximab [6,7]. The precise mechanism of thrombosis in ITP is unknown but has several explanations. First, it can be due to the presence of large immature prothrombotic platelets. These are mostly present during rituximab and IVIG therapy which induces an abrupt rise in the platelet count. Second, both platelets and endothelial cells can be targeted by anti-IIb/IIIa antibodies due to antigenic mimicry. Third, antiphospholipid antibodies are seen frequently in patients with ITP and have been reported to cause increased thrombosis [8]. Finally, patients with ITP are more prone to IVIG-related thrombotic arterial/venous complications including pulmonary embolism/deep venous thrombosis, myocardial infarction, and stroke [9,10].

Acute coronary syndrome (ACS) in patients with ITP represents a challenge in terms of therapeutic management. In their published review, Torbey *et al.*[5] found 22 admissions of patients with ACS and ITP. Their median age was 66 years; 47.36% were female and 52.64% were male. Of these, 25% presented as non-ST elevated myocardial infarction, 45% as ST elevated myocardial infarction, while 25% had elective procedures performed. The mean platelet count on presentation was 66±83×10^3^/mL. The PCI was performed in all cases except one. A femoral access was adopted in 80% of cases. Pretreatment with steroids, IVIG, and platelets was required in 52%, 27%, and 13% of cases, respectively. Danazol [5] was used in two cases and romiplostim in two other cases [5]. The platelet count at the time of intervention was only known in 15 patients. The mean platelet count at which PCI was performed was 145±87×10^9^/L. Major and minor bleeding occurred in 12% and 5% of cases, respectively. Heparin was used in 87% of cases (in six cases the anticoagulant agent was not mentioned). Hemostasis was achieved in 76.1% of cases by manual compression. No serious bleeding complications occurred as manual compression was used in most cases except for Park *et al.*[6] who used an occlusion device, the Angio-Seal™. The patients were on single or dual antiplatelet therapy in 83% of cases. The longest follow-up period while on platelet anti-aggregants was 6 months.

Our case is exclusive, because our patient is one of the rare patients who used IVIG.

Choosing the safest intravenous anticoagulant and platelet anti-aggregant is another concern that arises during angioplasty. Unfractionated heparin (UFH) has been used in most cases without any significant complication while fondaparinux has been proposed as an alternative to UFH. Unlike UFH and low-molecular-weight heparin, fondaparinux is a pure antithrombin III-dependent factor Xa inhibitor which has no effect on platelet function. This may potentially decrease the risk of serious bleeding in thrombocytopenic patients. Neskovic *et al.*[7] documented the successful use of half dose of UFH in addition to fondaparinux during PCI for ACS in an 80-year-old patient without bleeding complications.

The favorable outcome in the 80-year-old patient might suggest that this combination could be considered in patients with the highest periprocedural risk. Bivalirudin being a direct antithrombin is another alternative to heparin since it has been used in heparin-induced thrombocytopenia.

There are no clear data in the literature regarding GpIIb/IIIa use in ITP patients presenting with ACS. Stouffer *et al.*[10] described a case where the coadministration of clopidogrel and eptifibatide was not associated with significant bleeding. Yagmur *et al.*[8] have reported the safe administration of tirofiban that satisfactorily dissolved the thrombi, and the platelets remained constant at 54×10^9^/L without any bleeding event. However, these two case reports do not eliminate the high probability of bleed while on GpIIb/IIIa, and thus further prospective investigational studies are needed.

In our case, before the procedure, in analogy to the literature we have used UFH and dual antiplatelet treatment.

The type of stent chosen will also have significant impact on the risk of bleeding in patients with ITP. The type of stent will have an impact on the duration of dual platelet anti-aggregants administration as well as on the rate of restenosis. Among the cases found in the literature, only two cases deferred the deployment of a stent in the setting of ACS to avoid chronic dual antiplatelet therapy. Unfortunately, in both cases, reinfarction occurred which required urgent angioplasty [9-11]. Most experts recommend bare metal stent placement as opposed to drug-eluted stent to minimize the duration of dual antiplatelet therapy in case bleeding occurs [12-15]. In our case, our patient received a bare metal stent because of the bleeding risks; this decision was made after discussion with the Department of Hematology.

## Conclusions

In conclusion, patients with history of ITP or patients with a low platelet count presenting with ACS usually require a tailored medical and interventional management. Standardized therapy is recommended while the patient is in remission. Patients presenting with low platelets (<30×10^9^/L) or with active bleed should be considered for pretreatment with steroids and IVIG while postponing, if possible, the treatment of the coronary syndrome; if treatment is required, the option remains to use bivalirudin. The administration of half the dose of UFH with or without fondaparinux seems to be an alternative option for anticoagulation, but further studies are needed to prove the efficacy of such an antithrombotic approach. A coronary computed tomography angiogram can be considered prior to PCI to rule out a thrombotic cause of ACS.

Finally, dual antiplatelet therapy appears to be tolerated in patients with ITP, but caution should be undertaken to withhold at least one of them during an ITP relapse.

## Consent

Written informed consent was obtained from the patient for publication of this case report and any accompanying images. A copy of the written consent is available for review by the Editor-in-Chief of this journal.

## Abbreviations

ACS: Acute coronary syndrome; AMI: Acute myocardial infarction; ECG: Electrocardiography; Gp: Glycoprotein; ITP: Immune thrombocytopenic purpura; IVIG: Intravenous immunoglobulin; LAD: Left anterior descending; PCI: Percutaneous coronary intervention; UFH: Unfractionated heparin.

## Competing interests

The authors declare that they have no competing interests.

## Authors’ contributions

BD and MA participated in the design of study. MA, FB, MC and MG conceived of the study and participated in its design and coordination. BD participated in article writing. BE participated in correction of the study. All authors read and approved the final manuscript.
